# A Data Rate Monitoring Approach for Cyberattack Detection in Digital Twin Communication

**DOI:** 10.3390/s25247476

**Published:** 2025-12-09

**Authors:** Cláudio Rodrigues, Waldir S. S. Júnior, Wilson Oliveira, Isomar Lima

**Affiliations:** 1 Conecthus Institute—Technology and Biotechnology of Amazonas, Manaus 69075-000, Brazil; isomar.lima@conecthus.org.br; 2 CETELI—Center for Research and Development in Electronic Information Technology, Federal University of Amazonas—UFAM, Manaus 69067-005, Brazil; waldirjr@ufam.edu.br; 3 CTS—Superior Course of Technology, Ulbra Manaus—Universidade Luterana do Brasil, Manaus 69077-730, Brazil; woliveira1728@rede.ulbra.br

**Keywords:** digital twins, data rate, cyberattacks, IIoT security

## Abstract

The growing integration of Digital Twins (DTs) in Industry 4.0 environments exposes the physical–virtual communication layer as a critical vector for cyber vulnerabilities; while most studies focus on complex and resource-intensive security mechanisms, this work demonstrates that the inherently predictable nature of DT communications allows simple statistical metrics—such as the μ+3σ threshold—to provide robust, interpretable, and computationally efficient anomaly detection. Using a Docker-based simulation, we emulate Denial-of-Service (DoS), Man-in-the-Middle (MiTM), and intrusion attacks, showing that each generates a distinct statistical signature (e.g., a 50-fold increase in packet rate during DoS). The results confirm that data rate monitoring offers a viable, non-intrusive, and cost-effective first line of defense, thereby enhancing the resilience of IIoT-based Digital Twins.

## 1. Introduction

The adoption of Digital Twins (DTs) has become a cornerstone of Industry 4.0, transforming how complex systems are monitored, analyzed, and optimized in real time [1,2]. By creating dynamic, high-fidelity virtual replicas of physical assets or processes, DTs enable precise and proactive decision-making, with applications spanning from manufacturing to critical infrastructure management [3,4]. This technology operates through a continuous, bidirectional data stream that synchronizes the states of the physical and digital entities, ensuring real-time consistency.

Despite its operational advantages, this persistent interconnection introduces a novel and critical vector for cyber vulnerabilities. The communication layer, responsible for transporting data from Industrial Internet of Things (IIoT) sensors and Programmable Logic Controller (PLC) commands, becomes a primary attack surface where the integrity, confidentiality and availability of data can be compromised [5,6]. Attacks targeting this channel can corrupt the fidelity of the Digital Twin and may also trigger hazardous actions within the physical system, potentially resulting in severe operational disruptions and safety risks.

Recent studies have explored multiple strategies to mitigate these threats, including Machine Learning (ML)-based detection and blockchain-enabled authentication mechanisms [7,8,9]. However, as emphasized in our structured review (Section 3), these methods frequently impose substantial computational overhead, rendering them less practical for resource-constrained edge environments. Conversely, traditional low-cost defenses, such as static firewall rules, remain inadequate against novel (zero-day) attacks. This observation reveals a crucial research gap: the absence of detection mechanisms that are both computationally efficient and behaviorally resilient, specifically designed for the unique communication dynamics of IIoT-based Digital Twins.

To bridge this gap, the core contribution of this study is to demonstrate that the inherent regularity of IIoT and Digital Twin communication can serve as its own most effective line of defense. Unlike conventional IT networks, which exhibit irregular and stochastic traffic, IIoT environments are characterized by cyclical, deterministic, and highly predictable communication patterns—such as periodic sensor data reporting and command delivery to actuators. We argue that this predictability enables the use of lightweight, statistically grounded techniques exemplified by our non-intrusive approach based on the μ+3σ threshold. This statistical threshold corresponds to the mean plus three standard deviations and defines the upper boundary of normal behavior, enabling the detection of deviations that are statistically unlikely to occur under regular operating conditions. The findings of this work validate that data rate monitoring, when tailored to this specific context, constitutes a transparent, interpretable, and computationally efficient first layer of cyber defense.

The remainder of this paper is organized as follows. Section 2 presents the conceptual background for this work. Section 3 presents a critical review of the literature and defines the research gaps. Section 4 details the methodology used, including the detection approach and experimental setup. Section 5 presents and discusses the results obtained from the simulation experiments. Finally, Section 6 concludes the paper, summarizing the findings, discussing the limitations, and suggesting directions for future research.

## 2. Background

The transition to cyber-physical production systems enabled by Digital Twins relies critically on maintaining the integrity, confidentiality, and availability of the data streams that link the physical and virtual domains. This section provides the necessary conceptual background for this work. It first defines Digital Twin technology, architectures, and connectivity challenges. It then examines the broader cybersecurity landscape in Operational Technology (OT) and IIoT environments, concluding with a review of emerging threats that target these systems.

### 2.1. Digital Twins: Concepts, Architectures, and Connectivity Challenges

A Digital Twin (DT) is a dynamic, high-fidelity virtual representation of a physical asset, process, or system [1]. This digital model is continuously synchronized through real-time data collection from sensors and other sources in the physical environment, enabling it to replicate the behavior and characteristics of its physical counterpart for observation, analysis, and operational optimization [3]. A fundamental pillar of this technology is the bidirectional interaction between the physical domain and its digital counterpart: real-world data feed the virtual model, which, in turn, generates insights that influence the physical system, establishing a continuous cycle of control and improvement [1,10]. The implementation of DTs is facilitated by the convergence of technologies such as the Internet of Things (IoT), Artificial Intelligence (AI), and Machine Learning (ML) [11]. Their inherently connected nature and reliance on data exchange underscore the critical importance of communication security [5].

Building an effective Digital Twin (DT) requires a robust and well-defined architecture. Although various models have been proposed in the literature [12], a fundamental set of core components is commonly shared. Conceptually, a DT architecture can be structured into several key layers. The first layer is the physical entity, representing the real-world system and including the sensors and actuators responsible for data collection and interaction [5]. The second layer is the virtual model—a digital representation comprising components ranging from 3D models to AI algorithms used to process data, simulate scenarios, and optimize operations [11,13]. Serving as the bridge between these layers, the data connection layer enables the bidirectional flow of information, in which IoT and communication network technologies play a crucial role [5]. Finally, the service and application layer leverages the insights generated by the DT to deliver value through control dashboards, predictive maintenance tools, and decision-support systems [12].

The seamless integration of these layers facilitates the successful application of DTs across domains ranging from manufacturing to smart city management [3]. It is evident that the data connection layer functions as the central nervous system of the entire architecture. Its integrity and availability are not only critical functional requirements but also represent the primary potential attack surface. Therefore, understanding the security challenges inherent to this connectivity is a prerequisite for developing more resilient DT systems.

### 2.2. Emerging Cyber Threats and Their Impacts

Digital Twin (DT) security addresses not only known threats but also an ever-evolving attack landscape. The increasing sophistication of threats such as Advanced Persistent Threats (APTs) and AI-driven attacks—designed to remain stealthy and persistent—poses a particular challenge. Such attacks may not seek immediate service disruption but instead aim for continuous data exfiltration or the subtle, long-term manipulation of physical operations by corrupting the data stream that feeds the Digital Twin.

This new generation of threats tests the limits of existing detection mechanisms. Evasion attacks, for instance, are intentionally crafted to circumvent AI-based systems by subtly manipulating input data so that it is misclassified as benign traffic [14]. Moreover, these advanced attack capabilities exacerbate systemic concerns about data privacy and security in Digital Twins, potentially leading to large-scale data manipulation or critical information leakage, as discussed in [15].

The impact of these emerging threats on the physical–virtual communication flow is therefore direct. A sophisticated attack may not produce an obvious traffic spike, as in a DDoS event, but it can alter the frequency, size, or regularity of data packets, causing subtle deviations from the operational baseline. This underscores the need for monitoring approaches sensitive to fundamental variations in the communication channel.

## 3. Related Work

To contextualize the contribution of this work, we conducted a critical review of the state-of-the-art in DT intrusion detection. A structured and reproducible search methodology was developed and applied across major scientific databases, including IEEE Xplore and the ACM Digital Library. The search string employed was: (“digital twin” OR “digital twins”) AND (“intrusion detection” OR “attack detection” OR “anomaly detection” OR “threat detection” OR detection) AND (“industrial IoT” OR IIoT OR “industrial control system”). Following the initial retrieval, the resulting corpus was subjected to a multi-stage screening process designed to ensure both temporal relevance and thematic precision. Publications from 2020 onward were prioritized, with inclusion criteria focusing on the explicit occurrence of search terms within titles and abstracts. After applying these filters, a final corpus of 21 articles was selected, representing the most relevant state-of-the-art research at the intersection of Digital Twin technology and cyberattack detection. Table 1 summarizes the main features of these studies and serves as the analytical foundation for the discussion presented in the following subsections.

### 3.1. Intrusion Detection in the Physical–Virtual Communication of Digital Twins

The fidelity and utility of a Digital Twin (DT) are directly proportional to the integrity of its bidirectional communication with the physical counterpart. As highlighted previously, ensuring the Confidentiality, Integrity, and Availability (CIA) of this channel is imperative [5,6]. Any compromise of this data stream—whether through manipulation, interception, or disruption—can invalidate the virtual model and lead to erroneous or hazardous operational decisions in the physical environment [15]. Therefore, this subsection focuses on studies that directly address threat detection within this communication layer.

Recent literature proposes several strategies to mitigate these challenges. A major line of research employs machine learning and deep learning techniques for anomaly detection. For example, Liu et al. [7] explores the use of Federated Learning to train intrusion detection models in a distributed manner, thereby preserving data privacy. Others, such as [9], adopt a similar approach with a specific focus on detecting Distributed Denial-of-Service (DDoS) attacks in IIoT networks supporting Digital Twins. In parallel, there is growing interest in leveraging blockchain technology to ensure the integrity and traceability of exchanged data. Studies such as [8,16] propose architectures in which data transactions between the physical and virtual domains are immutably recorded, preventing undetected manipulation.

### 3.2. Literature Gaps and Proposed Contribution

A critical analysis of these contributions reveals a notable gap. Although effective against certain attack classes, many of these approaches introduce considerable computational and latency overheads (as in the case of blockchain) or require extensive labeled datasets for training (as with supervised deep learning). More importantly, few studies investigate low-level network metrics—such as throughput—as primary indicators of security anomalies. The hypothesis that subtle or abrupt variations in the volume and frequency of data packets can signal different types of attacks—ranging from DoS disruptions to reconnaissance or false data injection—remains relatively underexplored. This shortcoming justifies the investigation of a lightweight, computationally efficient detection method focused directly on the fundamental behavior of the communication channel.

The literature analysis reveals a vibrant research field with significant progress in defining Digital Twin (DT) architectures and applying techniques such as machine learning and blockchain to enhance security in IIoT environments. Despite these advances, our structured review highlights critical gaps that remain and motivate the present investigation. The main identified gaps are as follows:

- Architectures with reactive security: Most architectural models treat security as an external or adjunct layer rather than as an intrinsic design principle. There is a lack of proposals for architectures conceived from the outset to facilitate intrusion detection within communication flows while considering the performance constraints of IIoT systems.
- Focus on complex, high-level methods: Predominant detection strategies rely on computationally intensive approaches (e.g., deep learning, federated learning, blockchain) or on system-level behavioral analysis, often neglecting fundamental, low-cost communication channel metrics.
- Lack of throughput-focused detection: Specifically, the exploration of data throughput as a primary indicator for detecting a wide range of attacks—from service disruptions to subtle manipulations—remains notably underexplored in the Digital Twin the literature.
- Limited validation against emerging threats: There is a pressing need for experimental validation of detection strategies capable of operating in real time and maintaining DT resilience under sophisticated, stealthy attacks that target data integrity rather than merely causing denial of service.

In light of these gaps, this study proposes and validates an approach for detecting cyberattacks in the communication of industrial Digital Twins, based on monitoring and analyzing anomalies in data throughput. The proposed method aims to provide a non-intrusive, computationally lightweight, and complementary solution to existing strategies, thereby contributing to the development of more secure and resilient Digital Twin architectures within IIoT environments.

## 4. Methodology

This section details the methodology used to validate the proposed detection approach. It is structured to first explain the conceptual foundation of the method and then describe the experimental implementation.

### 4.1. Detection Approach

The central hypothesis of this work is that the inherent predictabilityof IIoT and Digital Twin communication makes it an ideal subject for statistical anomaly detection. Unlike traditional IT networks, which often exhibit irregular and stochastic traffic, DT environments are characterized by cyclical, deterministic, and highly predictable data flows (e.g., sensors reporting at fixed intervals).

Given this predictability, any significant deviation from a stable baseline can serve as a robust indicator of an anomaly. Our approach, therefore, is grounded in establishing this baseline and applying a lightweight, statistically rigorous threshold.

First, the benign (normal operation) workload was characterized to establish this baseline. Data rate metrics—Packets per Second (PPS) and Bytes per Second (BPS)—were recorded in one-second time windows. The mean (μ) and standard deviation (σ) of these metrics were then computed to establish the statistical baseline (as detailed in Table 2).

Second, anomaly detection is implemented based on this baseline. For each new data rate measurement collected during fault and attack scenarios, an anomaly is flagged whenever the observed value exceeds a defined threshold. As an initial criterion, a threshold of three standard deviations (μ±3σ) was adopted, a common practice in statistical process control for identifying statistically significant deviations. This “white box” method is computationally efficient, requires no training, and is highly interpretable, making it ideal for resource-constrained edge devices.

### 4.2. Experimental Setup and Implementation

The experimental environment was developed using Docker containerization technology. This platform was selected for its ability to create isolated, lightweight, and reproducible environments, making it ideal for simulating the complex architecture of a Digital Twin (DT) system. The simulation architecture consisted of three main container types, each fulfilling a specific role:

- Simulated physical device containers: These containers emulate the behavior of Programmable Logic Controllers (PLCs) and Industrial Internet of Things (IIoT) devices.
- Digital Twin container: This container hosts the virtual representation of the physical devices and functions as a system capable of receiving, processing, and storing data across various communication protocols.
- Monitoring container (Sniffer): Operating as a dedicated component, this container is responsible for capturing and analyzing network traffic without interfering with the primary data flow.

**Figure 1:**
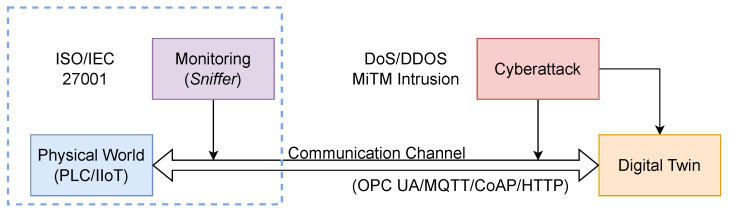
Conceptual architecture of the non-intrusive monitoring approach. The diagram illustrates the communication flow between the physical world (PLC/IIoT) and the Digital Twin, which relies on a communication channel (e.g., OPC UA/MQTT). This channel is identified as the primary attack vector for threats such as DoS, MiTM, and intrusion. The core component of our method, the “Monitoring (Sniffer),” is strategically positioned to non-intrusively capture data rate metrics from this channel for anomaly detection.

An overview of the proposed method is presented in Figure 1. The monitoring system (Sniffer) non-intrusively observes the communication channel that connects the physical world and its digital representation—the Digital Twin—to detect anomalies that may indicate a cyberattack.

The monitoring system (Sniffer) non-intrusively observes the communication channel linking the physical world (simulated by PLC and IIoT devices) and its digital representation—the Digital Twin (DT). The Sniffer continuously analyzes the data rate, expressed in Packets per Second (PPS) and Bytes per Second (BPS), to detect anomalies that may indicate a cyberattack. It operates within a conceptual security perimeter aligned with international standards such as ISO/IEC 27001 [34].

**Figure 2:**
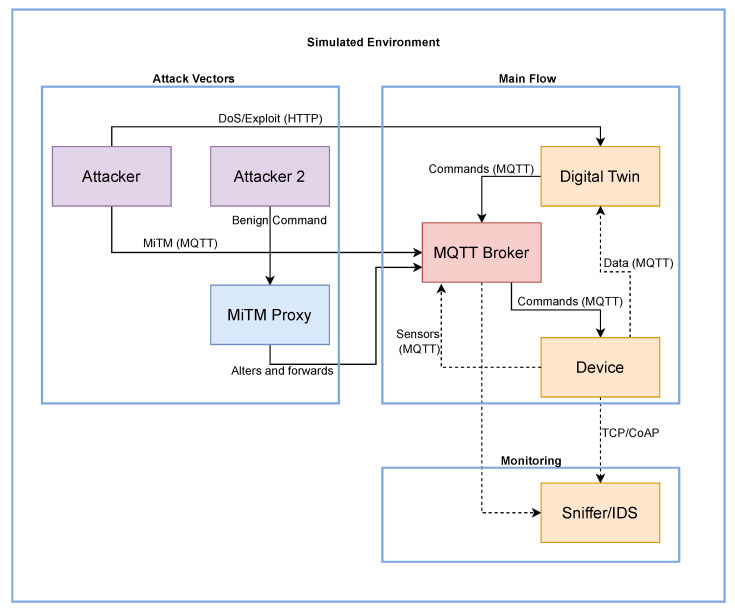
Logical architecture of the simulation environment. The flowchart details the experimental setup used to validate the proposed detection method. It illustrates the interactions among key components, including the simulated Device (sensors), the MQTT Broker, and the Digital Twin. Crucially, it identifies the injection points for attack vectors (DoS and MiTM) and the strategic placement of the “Monitoring (Sniffer/IDS)” component, which non-intrusively captures all network traffic for data rate analysis. In the diagram, solid arrows represent the active data and command communication flow, while dashed arrows indicate the passive traffic monitoring and analysis performed by the Sniffer/IDS.

It is important to clarify the relationship between the conceptual architecture (Figure 1) and the logical implementation (Figure 2). The conceptual method itself (Figure 1) is protocol-agnostic, as it monitors only data rates (PPS/BPS), not packet content, a strength we discuss in the Discussion (Section 5). For the experimental validation, however, a specific protocol stack had to be implemented. We selected MQTT as the primary communication channel for our logical architecture (Figure 2), as it is a dominant and representative standard in IIoT environments.

The detailed logical architecture of the simulation environment is shown in Figure 2. The diagram illustrates the interactions among the main containerized services, grouped into three functional domains: Main Flow, Monitoring, and Attack Vectors. The main operational flow (Main Flow) consists of data and command exchanges via MQTT between the Device and the Digital Twin, mediated by the MQTT Broker. The Monitoring component (Sniffer/IDS) passively observes this traffic, while the Attack Vectors represent the various entry points for simulated attacks, including direct assaults on the Digital Twin’s API and interception of MQTT traffic through a proxy (MiTM Proxy).

The experiments were conducted in a controlled environment. The host operating system was Ubuntu 24.04.3 LTS. Containerization was managed using Docker Engine version 28.4.0 and Docker Compose version 2.39.4. All scripts were developed in Python version 3.12. The system’s functionality relied on several key libraries, whose versions are essential for reproducibility: Paho-MQTT 1.6.1, Requests 2.32.3, Python-OPC-UA 0.98.13, and aiocoap 0.4.3. This documented configuration provides a solid foundation for replicating the experiments, enabling other researchers to reproduce and extend the proposed scenarios. The complete source code is available as detailed in the Data Availability Section.

### 4.3. Scenario Simulation and Validation

**Figure 3:**
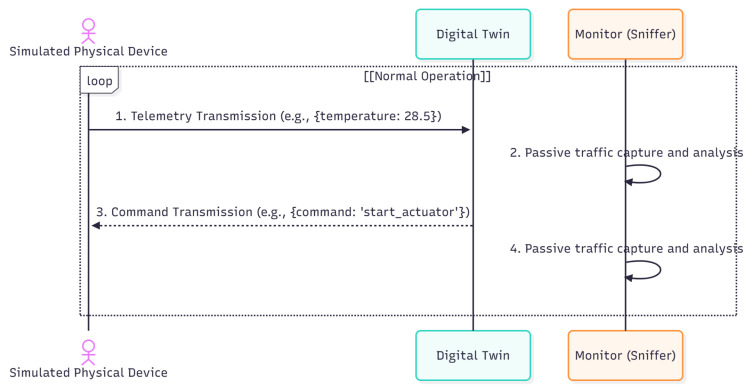
UML Sequence Diagram of the Non-Intrusive Monitoring Logic. This diagram depicts the standard MQTT publish/subscribe communication flow among the Device, MQTT Broker, and Digital Twin. It explicitly illustrates the role of the Monitoring (Sniffer) component, which operates in parallel to non-intrusively capture all network traffic. The Sniffer performs continuous data rate analysis (PPS/BPS) and, upon detecting a statistical anomaly (i.e., a threshold breach), triggers an alert to the Digital Twin, demonstrating the method’s passive detection capability. In the diagram, solid arrows represent the active data and command communication flow, while dashed arrows indicate the passive traffic monitoring and analysis performed by the Sniffer/IDS.

A fundamental step of our methodology involved simulating different operational scenarios to characterize the impact of anomalous events on transmission data rates. The objective was to establish a comparative basis for distinguishing anomalies resulting from malicious cyberattacks. As illustrated in the sequence diagram (Figure 3), the benign workload consists of the device container (simulating sensors such as temperature and humidity) periodically sending telemetry data via MQTT to the mqtt_broker, which then forwards it to the digital_twin. This stable, cyclical baseline traffic was recorded for 60 min without any faults or attacks. To this end, the following types of communication faults were simulated:

- Loss of connectivity: Simulation of temporary and permanent interruptions in the communication of specific devices or in the connection with the Digital Twin.
- Network latency: Introduction of variable network delays to observe their effect on response time and data transfer rates.
- Transmission errors: Simulation of packet transmission errors to induce retransmissions and observe the consequent variations in the data rate, mimicking potential data corruption.
- Bandwidth limitation: Introduction of artificial network bottlenecks to reduce transmission capacity and simulate congestion conditions.

For each type of fault, specific network control methods were applied using the Linux tc (traffic control) utility with netem (Network Emulator) within the Docker environment. For example, Network Latency was introduced by applying netem delay rules via the tc utility, and Bandwidth Limitation was simulated using a tbf (Token Bucket Filter). Parameters such as duration and intensity were varied based on a risk analysis and common faults observed in industrial and IIoT systems. The analysis of the data collected during these simulations enabled the identification of specific patterns used later to distinguish operational faults from cyberattacks.

To investigate the sensitivity of the data rate as an indicator of malicious activity, several types of cyberattacks were simulated, targeting both the communication between the simulated physical elements and the Digital Twin, and the Digital Twin itself. The main targets were the communication channel and the Digital Twin container, with the goal of compromising the integrity and availability of the exchanged data. The simulated attacks included:

- Denial-of-Service (DoS) and Distributed Denial-of-Service (DDoS): These attacks aim to render the system unavailable by overwhelming communication resources or the Digital Twin itself with excessive requests or malicious traffic. This was implemented using a custom Python script that leveraged multi-threading to generate high volumes of HTTP GET requests directed at the Digital Twin’s API.
- Man-in-the-Middle (MiTM): This attack seeks to intercept communication between the physical devices and the Digital Twin, with the potential to read or modify data in transit. This was simulated using a custom Python-based TCP proxy (as seen in Figure 2), which positioned itself at the communication layer to intercept traffic.
- Intrusion and asset compromise: This attack targets system vulnerabilities to gain unauthorized access to the Digital Twin, sending malicious commands (e.g., shutting down sensors) to the physical asset. This scenario, simulating an exploit, was implemented by sending malicious commands (e.g., `shut-down’) via unauthorized HTTP POST requests to the Digital Twin’s API, simulating a sabotage attack.

The intensity parameters (e.g., 50 concurrent threads for the DoS attack and a 5 s burst for the Intrusion scenario) were selected to support functional validation. We chose to simulate medium-to-high-intensity threats commonly observed in industrial environments, ensuring that the resulting anomalies clearly exceeded the μ+3σ statistical threshold. This validation strategy demonstrates the method’s effectiveness as a first line of defense against non-stealthy threats that compromise system availability and integrity.

For each attack type, parameters such as intensity and activity characteristics were varied. This allowed us to evaluate whether the anomalies in the data rate could be detected under different conditions and intensity levels, in order to determine detection thresholds and assess the sensitivity of the proposed method to diverse classes of threats.

The bidirectional communication flow during normal operation is illustrated in the sequence diagram shown in Figure 3. The simulated physical device sends telemetry data to the Digital Twin (step 1), which, in turn, can issue actuation commands to the device (step 3). The monitoring component (Sniffer), positioned within the communication channel, passively captures and analyzes all traffic (steps 2 and 4).

The third and final stage consisted of validating the methodology by verifying the correlation between the detected anomalies and the simulated events. This validation was performed by overlaying time series graphs, plotting the data rate alongside visual indicators marking the start and end of each fault or attack. This temporal analysis confirmed whether the detected anomalies coincided with the simulated events, thereby validating the method’s ability to accurately identify abnormal activities.

**Figure 4:**
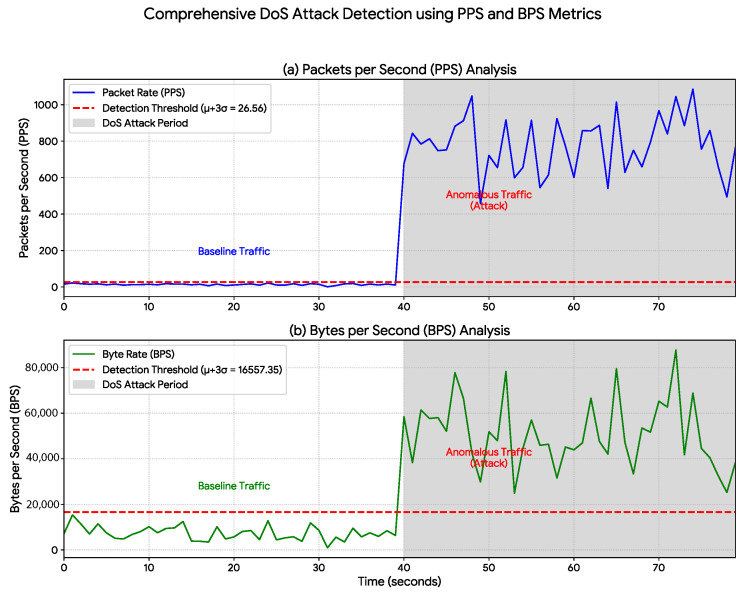
DoS Attack Detection Using PPS and BPS Metrics. Detection of a DoS attack (shaded region, t≥40 s) through data-rate monitoring. A baseline statistical threshold (red dashed line), corresponding to μ+3σ, was established based on normal operation (t<40 s). Upon attack initiation at t=40 s, both (**a**) Packets per Second (PPS) and (**b**) Bytes per Second (BPS) exhibit immediate and significant spikes that exceed the threshold, validating the method’s effectiveness in identifying high-volume attacks.

**Figure 5:**
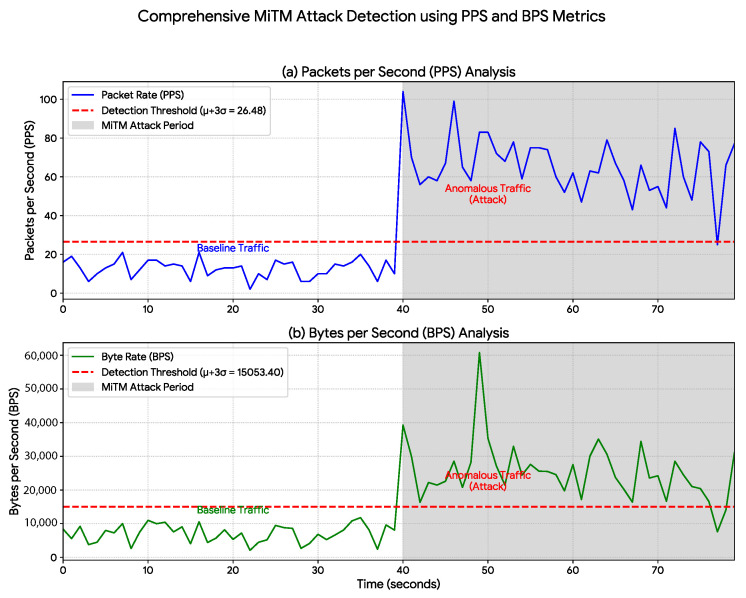
MiTM Attack Detection Using PPS and BPS Metrics. Detection of a Man-in-the-Middle (MiTM) attack initiated at t=40 s (shaded region). Although its statistical signature is more subtle than that of the DoS attack, the anomalous traffic promptly exceeds the established μ+3σ threshold (red dashed line) for both (**a**) Packets per Second (PPS) and (**b**) Bytes per Second (BPS). This result highlights the method’s sensitivity in identifying attacks that attempt to blend with normal traffic patterns.

**Figure 6:**
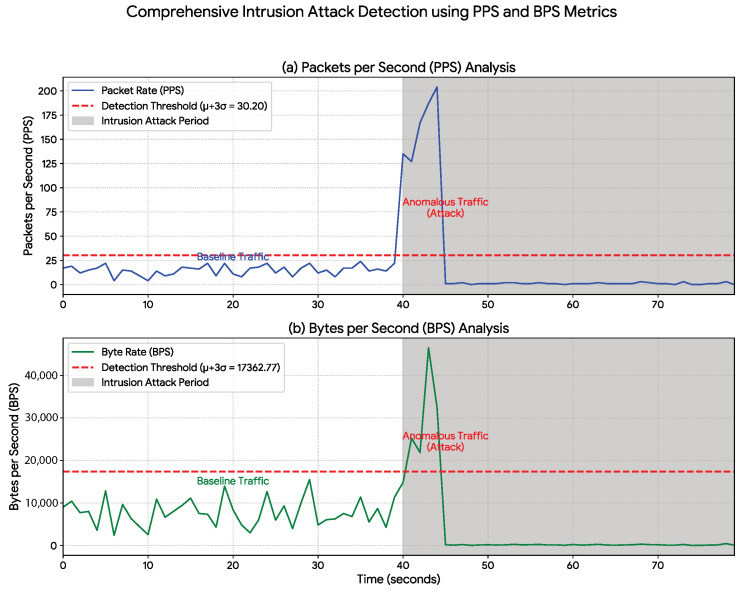
Intrusion Attack Detection Using PPS and BPS Metrics. Results from the intrusion attack simulation (shaded region, t≥40 s). The attack produces a distinct, short-duration traffic spike that markedly exceeds the μ+3σ statistical baseline (red dashed line) for both (**a**) Packets per Second (PPS) and (**b**) Bytes per Second (BPS). This clear threshold violation confirms the method’s capability to identify unauthorized intrusion events based on their data-rate footprint.

The attack scenarios presented in Section 5 were executed under the following parameters: the Denial-of-Service (DoS) attack (Figure 4) was generated using 50 concurrent threads, resulting in an increased packet rate averaging approximately 800 PPS; the Man-in-the-Middle (MiTM) attack (Figure 5) was modeled to simulate the overload caused by a malicious proxy, increasing the packet rate to an average of 60 PPS; and the Intrusion attack (Figure 6) consisted of a 5 s burst of activity, with packet rates peaking at 150 PPS to simulate the sending of sabotage commands.

## 5. Results and Discussion

This section presents the experimental results obtained and discusses their implications. Section 5.1 first describes the quantitative outcomes of the simulations, demonstrating the effectiveness of the proposed data-rate monitoring approach in detecting the three attack scenarios. Section 5.2 then provides a comparison with state-of-the-art (SOTA) methods. Subsequently, Section 5.3 interprets these findings, situating the contributions of this work within the existing literature, highlighting its advantages, and acknowledging its limitations.

### 5.1. Results

The proposed data-rate monitoring approach was evaluated across three distinct cyberattack scenarios. The results show a strong correlation between the simulated attacks and statistically significant anomalies in network-traffic metrics, thereby validating the method’s effectiveness. A detailed statistical analysis is presented in Table 2, while the temporal behavior of the traffic is illustrated in Figure 4, Figure 5 and Figure 6.

As shown in Table 2, network traffic under normal operating conditions exhibited a stable and consistent baseline across all experiments, with an average packet rate ranging between 12.70 and 14.95 PPS. This baseline served as a reliable reference for detecting deviations.

In the first scenario, the initiation of the Denial-of-Service (DoS) attack (Figure 4) resulted in the immediate detection of a drastic anomaly. Table 2 quantifies this event: the average packet rate increased from a baseline of 13.75 PPS to 779.30 PPS—a surge of over 50-fold. The plots clearly illustrate this traffic flood, where both packets per second (a) and bytes per second (b) escalate sharply to sustained extreme levels, confirming the method’s reliability in identifying volumetric attacks.

The Man-in-the-Middle (MiTM) attack (Figure 5) exhibited a more subtle yet clearly detectable signature. The average packet rate increased from 12.70 PPS to 65.68 PPS (Table 2). Although less pronounced than the DoS attack, this anomaly demonstrates the system’s sensitivity in detecting not only flooding events but also traffic manipulations indicative of communication interception.

The intrusion attack (Figure 6) exhibited a distinctive statistical signature. As shown in Table 2, although the mean PPS showed only a modest increase (from 14.95 to 21.52), the standard deviation rose sharply from 5.08 to 55.55. This high variance corresponds to the behavior depicted in the plot: a brief, high-intensity traffic peak (command injection) followed by an almost complete collapse in communication. These results demonstrate that the method can detect anomalies not only in traffic volume but also on instability and red abrupt state changes within the communication channel.

### 5.2. Comparison with State-of-the-Art Methods

A critical analysis of the literature necessitates comparing the proposed method with state-of-the-art (SOTA) approaches, particularly those based on Machine Learning (ML) and Deep Learning (DL), which have been extensively studied (as summarized in Table 1).

Table 3 synthesizes this comparison, focusing on two key metrics essential for a fair assessment: detection performance and computational efficiency (overhead).

It is important to note, as indicated by the asterisk (*) in Table 3, that the “Detection Performance” metrics of the SOTA methods and the proposed approach are not directly comparable. The SOTA values (e.g., 99.98%) typically refer to classification accuracy achieved on static benchmark datasets (e.g., KDD, CIC-IDS) following extensive training and feature extraction phases.

In contrast, the 100% value reported for our method represents the detection success rate within the simulated attack scenarios (Figure 4, Figure 5 and Figure 6). Notably, this success rate (True Positive Rate) was achieved with a 0% False Positive Rate (FPR) during the normal operation period. As shown in Figure 4, Figure 5 and Figure 6, the baseline traffic (t<40 s) consistently remained below the μ+3σ detection threshold. This distinction is critical: while SOTA approaches focus on complex traffic classification, our method—achieving 100% success in tests—emphasizes efficient, real-time detection of anomalous events in the IIoT context.

The analysis of Table 3 reveals a fundamental trade-off:

1. Accuracy vs. Complexity: SOTA methods such as [9,31] indeed achieve very high detection accuracies, often exceeding 99.5% in classifying anomalous traffic. However, this performance entails significant computational overhead, requiring intensive training, complex preprocessing and feature extraction, and frequently specialized hardware (e.g., GPUs) for real-time inference—requirements our method eliminates, as demonstrated in Section 4 and validated in Figure 4, Figure 5 and Figure 6.
2. Efficiency in IIoT Contexts: This high computational overhead makes complex ML/DL models impractical or undesirable in many IIoT and Digital Twin scenarios, where devices such as PLCs or edge sensors possess limited resources.

#### Strengths and Limitations

The principal strength of our approach, as supported by Table 3, lies in its exceptional computational efficiency. Calculating a μ+3σ threshold is a straightforward statistical operation that can be executed on a lightweight network sniffer or edge device with minimal resource consumption and without adding latency to the system.

The main limitation of the approach is its specialization in detecting anomalies that affect data-rate metrics. However, as demonstrated in Figure 4, Figure 5 and Figure 6, a wide range of attacks (DoS, MiTM, and Intrusion) generate distinct and detectable statistical signatures within these metrics.

In conclusion, although SOTA ML/DL methods may achieve higher accuracy in classifying complex attack types, the proposed low-cost statistical approach offers a more efficient, interpretable, and context-aware solution for Digital Twin security—serving as a robust first line of defense.

### 5.3. Discussion

The presented results provide compelling evidence supporting the viability of data-rate analysis as a primary indicator for detecting cyberattacks in Digital Twin (DT) communication channels. However, the significance of these findings extends beyond simple anomaly detection and can be interpreted through three complementary perspectives: the diagnostic versatility of the method, its operational implications for industrial environments, and its role as a lightweight and robust security mechanism.

The central implication of this study lies in demonstrating that fundamental network metrics—such as packet and byte rates—serve not only as performance indicators but also as valuable sources of security information. The method’s ability to detect three fundamentally distinct attack classes—a volumetric attack (DoS), an interception attack (MiTM), and a sabotage attack (Intrusion)—attests to its robustness and generalizability. Rather than being a threat-specific solution, this approach functions analogously to a “nervous system” for the communication channel, exhibiting sensitivity to multiple types of disturbances.

Each attack produced a distinct statistical signature, as detailed in Table 2. The DoS attack manifested as a substantial increase in mean PPS, while the MiTM attack exhibited a more moderate rise. The Intrusion attack, in contrast, was characterized not by an increase in the mean but by a sharp rise in variance (standard deviation). This observation is crucial: data-rate monitoring provides not only a binary alert (normal vs. anomalous) but also a foundation for preliminary threat characterization and classification, offering an operational advantage for prioritizing incident response.

This ability to support threat characterization underscores a fundamental contrast with many state-of-the-art (SOTA) approaches in the literature; while machine learning (ML) and deep learning (DL) methods have demonstrated high proficiency in detecting complex anomalies, they often entail significant computational costs [9,17,23,27,31] and suffer from limited interpretability—the so-called “black box” problem [9,26,31]. In Operational Technology (OT) environments, where computational resources are limited and rapid, transparent diagnostics are essential, AI-based alerts lacking causal explanations can be problematic. Operators need not only to know that an anomaly has been detected, but also why it was detected, as the underlying rationale determines the appropriate incident response.

It is important to clarify why these attacks generated these signatures. The MiTM attack (Figure 5) was not a passive eavesdropping attack, but an active TCP proxy; while the data volume might be similar, the proxy implementation inherently introduces significant protocol overhead (e.g., additional packet handshakes, acknowledgments, and proxy processing), which results in the moderate but clearly detectable increase in PPS and BPS. The Intrusion scenario (Figure 6) was simulated as a high-intensity burst of unauthorized HTTP POST commands designed for rapid sabotage. This burst (and not a "low-and-slow" attack) is what creates the distinct, short-duration, high-volume spike that our method detected.

In contrast, the approach proposed in this work is grounded in the principle of direct interpretability—a “white box” design. The cause of each alert is explicitly tied to a violation of a statistical threshold in fundamental and intuitive metrics, such as packets or bytes per second. This simplicity constitutes a strategic advantage rather than a limitation. The modest computational requirements for calculating means and standard deviations in real time make the method suitable for deployment on edge devices or resource-constrained gateways, serving as an efficient, low-latency first line of defense. This approach therefore directly addresses the trade-off identified in Table 3, prioritizing high adequacy and low overhead for IIoT contexts over the costly, high-precision classification of SOTA methods.

Additionally, the proposed approach is largely application-protocol agnostic; while many Intrusion Detection Systems (IDS) rely on specific parsing rules for protocols such as MQTT, OPC UA, or HTTP [35], data-rate monitoring operates at a lower abstraction level, remaining sensitive to anomalies regardless of the communication protocol employed. This grants greater flexibility and adaptability, especially in heterogeneous industrial ecosystems where legacy and modern technologies coexist.

It is crucial to consider potential adversarial strategies targeting a fixed statistical defense. Because the μ+3σ threshold is static, an attacker aware of this mechanism could perform a low-and-slow attack, gradually injecting data volumes just below the established threshold over an extended period; while the proposed method is highly effective against high-volume threats that compromise system availability, it is inherently limited in detecting subtle evasion attempts that favor stealth over speed. This limitation underscores the trade-off between the computational efficiency of the μ+3σ approach and its resilience against sophisticated, stealth-oriented attacks.

## 6. Conclusions

This study demonstrates the viability of a non-intrusive and computationally lightweight approach for detecting cyberattacks in Digital Twin (DT) communication channels—an increasingly critical threat vector in Industrial Internet of Things (IIoT) environments. Through a controlled simulation environment, we demonstrated that monitoring and statistically analyzing fundamental network metrics—specifically packet rate (PPS) and byte rate (BPS)—is highly effective for identifying a diverse range of malicious activities. The method proved robust, achieving a 100% Detection Success Rate (True Positive Rate) across the three simulated attack classes (DoS, MiTM, and Intrusion), with a 0% False Positive Rate (FPR) during normal operation (baseline).

The principal contribution of this work lies in validating that data-rate analysis can serve as a robust and interpretable first line of defense. More importantly, the study highlights that the intrinsically predictable and deterministic nature of IIoT traffic enables a simple statistical threshold (μ+3σ) to act as a high-fidelity anomaly detector. This approach addresses a critical gap in the literature, which largely focuses on computationally intensive methods (e.g., ML/DL), by offering a low-overhead alternative ideally suited for this context. The results indicate that this strategy can significantly enhance the resilience and security of Digital Twin systems by providing an effective early-warning mechanism.

In practical terms, this method can be deployed as a lightweight network sensor that monitors traffic at critical points within OT/IT networks. The generated anomaly alerts can be seamlessly integrated into existing Security Information and Event Management (SIEM) platforms, thereby enriching situational awareness without overburdening the control infrastructure. Future work will focus on validating the approach in physical testbeds and exploring adaptive thresholding techniques to further improve detection sensitivity.

Despite its effectiveness and advantages, certain limitations must be acknowledged, which also define opportunities for future research. The primary limitation arises from the experimental setup, as validation was performed within a controlled Docker-based simulation. Although this approach ensures reproducibility and controlled variable isolation, it cannot fully capture the complexity and unpredictability of physical industrial networks, including hardware jitter, electromagnetic interference, and the heterogeneous behavior of legacy components.

Furthermore, while the tested attack scenarios encompass distinct threat classes, they are not exhaustive. More sophisticated threats—such as low-and-slow or Advanced Persistent Threats (APT)—may induce data-rate anomalies that remain below simple statistical thresholds. Detecting such stealthy attacks will require more sensitive and adaptive analytical strategies beyond fixed-threshold monitoring.

Building upon these findings, future research can advance in three main directions. The first is the validation of the proposed method in physical testbeds integrating industrial hardware such as programmable logic controllers (PLCs), sensors, and network switches to evaluate real-world performance. The second involves expanding the detection scope by assessing sensitivity to a broader range of stealthy or evolving threats. Finally, the third direction entails exploring hybrid architectures, in which data-rate monitoring serves as a low-cost preliminary trigger that activates more computationally intensive analyses—such as deep packet inspection or ML-based classifiers—upon the detection of an initial anomaly, enabling an efficient, layered, and resilient defense system.

Overall, the proposed statistical data-rate monitoring approach offers a practical pathway toward more interpretable, efficient, and resource-aware cybersecurity in Industrial Digital Twin systems.

## Figures and Tables

**Table 1:** Structured Review of Anomaly-Detection Methods in IIoT and Digital Twins. This table summarizes key studies in the field, emphasizing the prevailing reliance on complex, high-overhead approaches (e.g., ML/DL). The review highlights the research gap addressed in this paper—the experimental validation of lightweight, data-rate-based detection methods.

Reference	System Type	Security Focus	Detection	Monitor Metric	Technique	Attacks	Validation
[9]	IIoT	D, P	AN	DR, SC	FL, DL	DDoS	SIM
[7]	IIoT	P, I	TD	N/A	FL, RS	GA	RS
[16]	IIoT	D, I	CA	NF	BC, ML	GA	SIM
[17]	IIoT	D, I	AN	NF	ML	CA	TB
[18]	ICS, IIoT	I, A	IDS	NF	DL, CNN, RNN	CA	RS
[19]	ICS, CPS	A, I	AD	SC	CT	CA	RS
[20]	CPS	D	AN	SC	DL, RS	GA	RS
[8]	IIoT	I, R, A	N/A	N/A	BC, RS	GA	RS
[21]	IS	D	FD, AN	SC	DL, DT	FA	DS
[22]	IIoT	A, I	AD	NF	FO	BT	TB
[23]	ICS	I, A	AD	SC	DT	CA	TB
[24]	IIoT	I, A	IDS	CF	DL, HY	MA	DS
[25]	IIoT	D, I	AN	SC	DT, RS	GA	RS
[26]	IoT	A, I	IDS	NF	DL, DNN	CA	TB
[27]	IoT	A, I	AD	NF	ML, RF	CA	TB
[28]	IoT	D, I	IDS	NF	DL, RNN	CA	TB
[29]	IoT	A, I	AN	NF	DL, DAE	BT	TB
[30]	ICS	A, I	TD	SC	RS	CA	RS
[31]	CPS	C, P	CA	SC	DL, FL	IN	SIM
[32]	IoT	C, A	TM	SC	RS	GA	RS
[33]	IS	C, I, A	TD	SC	AI, DT	GA	RS

System Type: IIoT (Industrial Internet of Things), ICS (Industrial Control System), CPS (Cyber-Physical System), IS (Industrial), IoT (Internet of Things). Security Focus: D (Availability), P (Privacy), I (Integrity), A (Authenticity), R (Resilience), C (Confidentiality). Detection: AN (Anomaly), TD (Threat Detection), CA (Collab. Anomaly), IDS (Intrusion Detection System), AD (Attack Detection), N/A (Not Applicable), FD (Fault Detection), TM (Trust Management). Monitoring Metric: DR (Data Rate), SC (System Comp.), N/A (Not Applicable), NF (Network Flow), CF (Code/File). Technique: FL (Federated Learning), DL (Deep Learning), RS (Research Survey), BC (Blockchain), ML (Machine Learning), CNN (Convolutional Neural Network), RNN (Recurrent Neural Network), CT (Control Theory), DT (Digital Twin), FO (Forensics), HY (Hybrid), DNN (Deep Neural Network), RF (Random Forest), DAE (Deep Autoencoder), AI (Artificial Intelligence). Attacks: DDoS (Distributed Denial-of-Service), GA (Generic Attacks), CA (Cyberattacks), FA (Faults), BT (Botnet), MA (Malware), IN (Intrusions). Validation: SIM (Simulation), RS (Review/Survey), TB (Testbed), DS (Dataset).

**Table 2:** Statistical Baseline Parameters for Anomaly Detection. Descriptive statistics—mean (μ) and standard deviation (σ)—for the normal-operation baseline (t<40 s) across all three simulated attack scenarios (DoS, MiTM, and Intrusion). These baseline values form the basis for computing the μ+3σ statistical thresholds used to detect anomalous traffic, as visualized in Figure 4, Figure 5 and Figure 6.

Attack Type	Period	Mean PPS	Std. Dev. PPS	Mean BPS	Std. Dev. BPS
DoS	Normal	13.75	4.27	7309.92	3082.47
DoS	Attack	779.30	156.03	50,904.75	15,049.37
MiTM	Normal	12.70	4.59	7085.28	2656.04
MiTM	Attack	65.68	14.97	25,445.80	8674.89
Intrusion	Normal	14.95	5.08	7721.48	3213.76
Intrusion	Attack	21.52	55.55	3664.50	10,141.59

**Table 3:** Comparison with State-of-the-Art (SOTA) Anomaly-Detection Methods in IIoT. This table contrasts the proposed low-overhead statistical approach (μ+3σ threshold) with state-of-the-art methods based on Machine Learning (ML) and Deep Learning (DL) identified in the literature. The comparison focuses on detection performance and computational efficiency, highlighting the trade-off between model complexity and suitability for resource-constrained IIoT environments.

Reference	SOTA Technique	Performance Metric	Computational Cost (Overhead)	Suitability for IIoT/Edge
[9]	FL, DL	99.98% (Accuracy)	High	Low
[31]	DL, FL	99.20% (Accuracy)	High	Low
[27]	ML	99.34% (Accuracy)	Medium	Low
[26]	DL	98.70% (Detection Rate)	High	Low
[17]	ML	99.98% (Accuracy)	Medium	Medium-Low
This paper	SDRM	100% (Detection Rate) *	Very Low	High

SOTA Technique: FL (Federated Learning), DL (Deep Learning), ML (Machine Learning), SDRM (Statistical Data Rate Monitoring). Computational Cost (Overhead): High: Refers to DL methods (FL, GANs, CNN-LSTM, Graph Models) that require intensive training and/or specialized hardware (GPU). Medium: Refers to ML methods (GCN, Multiples) that require training and significant feature extraction. Very Low: Refers to the proposed method, which requires no training and uses only simple statistical operations. Suitability for IIoT/Edge: Low indicates impracticality for deployment due to high computational demands. Medium-Low indicates a reduced practicality for deployment on edge devices due to high resource requirements, but maintains some feasibility (e.g., deployment on a powerful gateway). High indicates high adequacy for resource-constrained edge environments. * The 100% metric represents the Detection Success Rate of the method in the three simulated attack scenarios (DoS, MiTM, Intrusion), where the statistical threshold was violated in all events.

## Data Availability

The complete source code, Docker configuration files (*docker-compose.yml*), and scripts used to generate the results presented in this article are publicly available in a GitHub repository at the following address: https://github.com/woliveira1728/digital-twin/tree/main/digital_twin, accessed on 21 November 2025.

## Abbreviations

The following abbreviations are used in this manuscript:

DT	Digital Twin
DTs	Digital Twins
IIoT	Industrial Internet of Things
OT	Operational Technology
DoS	Denial-of-Service
MiTM	Man-in-the-Middle
PPS	Packets Per Second
BPS	Bytes Per Second
SIEM	Security Information and Event Management
APT	Advanced Persistent Threat
IDS	Intrusion Detection System
CIA	Confidentiality, Integrity, and Availability
PLC	Programmable Logic Controller
